# SiRNA Crosslinked Nanoparticles for the Treatment of Inflammation‐induced Liver Injury

**DOI:** 10.1002/advs.201600228

**Published:** 2016-09-02

**Authors:** Yaqin Tang, Ziying Zeng, Xiao He, Tingting Wang, Xinghai Ning, Xuli Feng

**Affiliations:** ^1^ Innovative Drug Research CenterChongqing UniversityChongqing401331P. R. China; ^2^ Department of Biomedical EngineeringNanjing UniversityNanjing210093P. R. China

**Keywords:** acid labile, copper free click, positive‐charge‐free, siRNA crosslinking, targeting

## Abstract

RNA interference mediated by small interfering RNA (siRNA) provides a powerful tool for gene regulation, and has a broad potential as a promising therapeutic strategy. However, therapeutics based on siRNA have had limited clinical success due to their undesirable pharmacokinetic properties. This study presents pH‐sensitive nanoparticles‐based siRNA delivery systems (PNSDS), which are positive‐charge‐free nanocarriers, composed of siRNA chemically crosslinked with multi‐armed poly(ethylene glycol) carriers via acid‐labile acetal linkers. The unique siRNA crosslinked structure of PNSDS allows it to have minimal cytotoxicity, high siRNA loading efficiency, and a stimulus‐responsive property that enables the selective intracellular release of siRNA in response to pH conditions. This study demonstrates that PNSDS can deliver tumor necrosis factor alpha (TNF‐α) siRNA into macrophages and induce the efficient down regulation of the targeted gene in complete cell culture media. Moreover, PNSDS with mannose targeting moieties can selectively accumulate in mice liver, induce specific inhibition of macrophage TNF‐α expression in vivo, and consequently protect mice from inflammation‐induced liver damages. Therefore, this novel siRNA delivering platform would greatly improve the therapeutic potential of RNAi based therapies.

## 1 Introduction

Small interfering RNA (siRNA) mediated RNA interference is a powerful tool for selective silencing of specific gene expression, and, therefore, has emerged as a promising therapeutic strategy against human diseases, such as cancer, autoimmune disorder, and viral infections via down regulating pathogenic genes.^1^, ^2^, ^3^ However, siRNA based therapeutics have had limited clinical success due to their undesirable pharmacokinetic properties including inherent instability, fast renal clearance, and poor cell permeability. Hence, the establishment of an effective and versatile siRNA delivery system is a key issue for achieving the clinical potential of siRNA.

Nanoparticulate nonvirial delivery platforms are highly attractive for siRNA therapy owing to a number of unique properties that can overcome various challenges and obstacles to the delivery of siRNA, particularly, bioavailability, and biodistribution.^4^ Numerous siRNA carriers based on nanotechnology have been developed, and most of them utilized non‐covalent association of siRNA with lipid complexes,^5^, ^6^, ^7^ conjugated polymers, magnetic nanoparticles, carbon nanotubes, gold nanoparticles, and quantum dot nanoparticles.^8^, ^9^, ^10^, ^11^, ^12^ However, these non‐covalent strategy based nanocarriers have various disadvantages due to their composition, physical, and chemical characteristics, thus leading to a range of incompetence when associated with siRNA. Up to date, the ideal nanoparticulate systems for siRNA delivery are still pursued by researchers, yet there remain numerous challenges associated with the siRNA delivery process.

Alternative methods for the formation of nanostructured siRNA delivery systems are based on chemical modifications of siRNA with lipids, small molecules, peptides, etc.^13^, ^14^, ^15^, ^16^ In particular, covalently crosslinked nanoparticles, known as nanoparticles, are a promising approach for stabilizing siRNA delivery systems in systemic circulation, and have received growing attention as a new class of siRNA carriers.^17^, ^18^, ^19^, ^20^ However, extensive crosslinking of nanoparticles also inhibits the release of siRNA from the complexes. To address this issue, crosslinked nanoparticles were designed to degrade in response to the intracellular and extracellular environment, such as reductive molecules, reactive oxygen species, temperature change, and pH reduction. Therefore, environmental stimulus‐sensitive crosslinked nanoparticles are promising candidates for maximizing the therapeutic efficacy of siRNA.

Currently, most popular environment‐responsive crosslinking methods used to covalently attach siRNA to nanoparticles are the disulfide bond formation. The disulfide linkages could react with intracellular reductive molecules, such as glutathione (GSH), which is an important antioxidant and can reduce disulfide bonds by serving as an electron donor. Crosslinked siRNA nanoparticles were prepared by crosslinking the siRNA bearing a terminal sulfanyl group with the polymer chains through the disulfide linkages.^21^, ^22^, ^23^, ^24^, ^25^, ^26^ The disulfide linkages are selectively cleaved by a sulfanyl‐disulfide exchange reaction with intracellular GSH, thereby allowing the release of the siRNA in the target cells. However, the nature of disulfide bond forming reaction, such as slow reaction rate, premature degradation, susceptibility of thiols to oxidation, cytotoxicity, and lack of bioorthogonality, has limited the potential application of the disulfide cross‐linked nanoparticles on systemic siRNA delivery.

**Figure 1:**
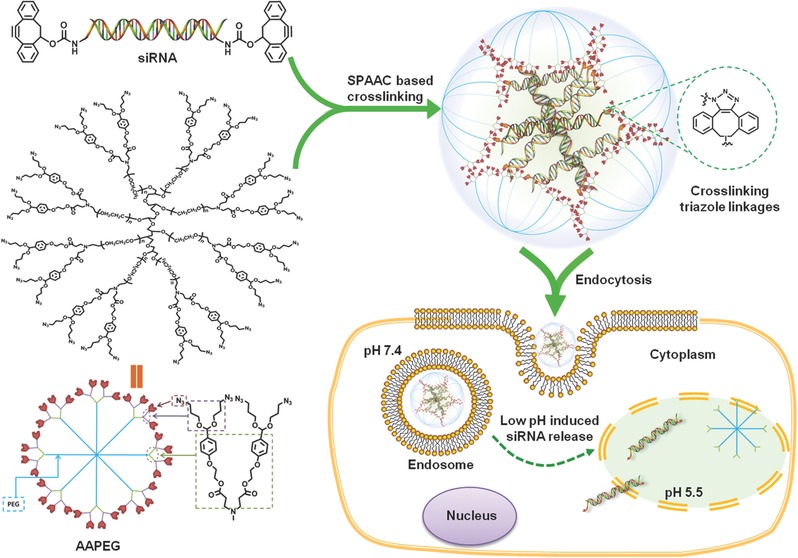
Schematic representation of siRNA cross‐linked nanoparticles (PNSDS).

Comparing to the intracellular reductive environment, microenvironmental pH variation is one of useful stimuli for altering the property of siRNA carriers and promoting the release of siRNA.^27^, ^28^, ^29^ Since siRNA carriers have to enter cells by the endocytic internalization, the pH of intracellular compartments after endocytosis, such as endosomes and lysosomes, is reduced to 4.5–6.5 from the extracellular pH 7.4. Therefore, we present a novel pH‐responsive nanoparticle system for improving the endosomal escape and the release of cross‐linked siRNA in cells. This siRNA crosslinked nanoparticle termed the PNSDS, is designated to selectively deliver siRNA in vivo, and silence gene expression in target cells. The chemical structure of PNSDS and its mechanism of intracelluar delivery of siRNA are shown in the **Figure** **1**. The PNSDS is composed of chemically modified siRNA that can crosslink to multi‐arm PEGs via hydrophobic acid‐labile acetal linkages,^30^ which contain endosome disrupting chain “capped” by hydrophilic siRNA and targeting residues.

The unique siRNA‐crosslinked structures of PNSDS allow it to improve siRNA stability, loading efficacy, cellular uptake, endosomal escape, and biological activity, endowing it with ability to mediate efficient gene silencing. siRNA is chemically crosslinked into the compact nanoparticle without the aid of any polycationic agents, and thus reduce the risk of nonspecific protein absorption, immunogenicity, and cytotoxicity. In addition, the tethering and tunable release of siRNA from PNSDS has also been achieved by the incorporation of acid labile acetal linkages near siRNA cross‐links, allowing PNSDS to release siRNA in response to the intracellular pH reduction. Furthermore, PNSDS has low cytoxicity, long blood circulation time, and minimal non‐specific interactions with serum proteins and non‐target tissues because of its positive‐charge‐free feature and protective PEG shell respectively. Finally, PNSDS is easy to be modified with targeting moieties, facilitating the target delivery of siRNA and inducing specific inhibition of gene expression in vivo. Therefore, the PNSDS would be an ideal siRNA delivery nanocarrier for efficient siRNA silencing.

## 2 Results and Discussion

### 2.1 Preparation of AAPEG

**Figure 2:**
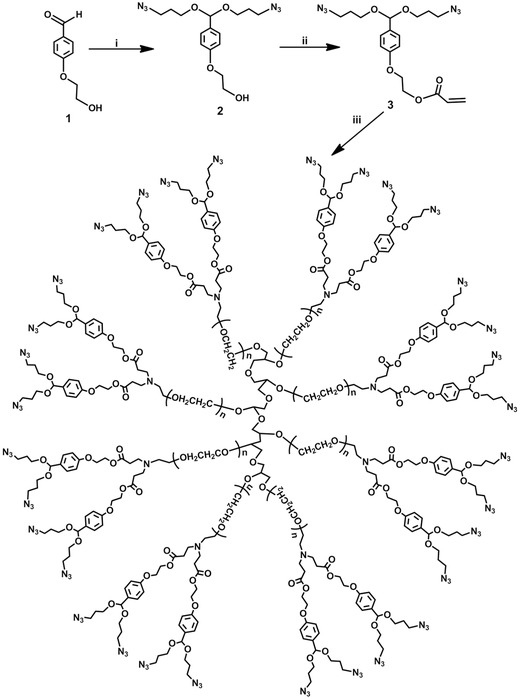
Synthetic routes of AAPEG. i) azidopropanol, PTSA, 5Å molecular sieves; ii) acrylate chloride, Et_3_N; ii) eight‐armed PEG amine, Et_3_N.

The most common materials chosen for the crosslinked nanoparticles is polyethyleneglycol (PEG) due to its excellent biocompatibility, low toxicity, and non‐adhesion toward protein and cells, and ability to decrease renal clearance. In this research, eight‐armed PEG functionalized with azide moieties (AAPEG) was clicked to cyclooctyne‐containing siRNA crosslinkers via copper‐free strain‐promoted azide‐alkyne cycloaddition (SPAAC),^31^ leading to the formation of crosslinked nanogles. AAPEG was synthesized using a direct Michael Addition of commercially available eight‐arm PEG‐NH2 with compound (3), which was synthesized through a two‐step reaction involving the aldolization of 4‐(2‐hydroxyethoxy) benzaldehyde (1) with 3‐azidopropan‐1‐ol followed by acrylation (shown in the **Figure** **2**). Subsequently, upon mixing AAPEG with siRNA crosslinkers, the SPAAC commenced and thus the crosslinked PNSDS formed, without the need of additional chemicals or further processing.

### 2.2 Preparation and Characterization of PNSDS

**Figure 3:**
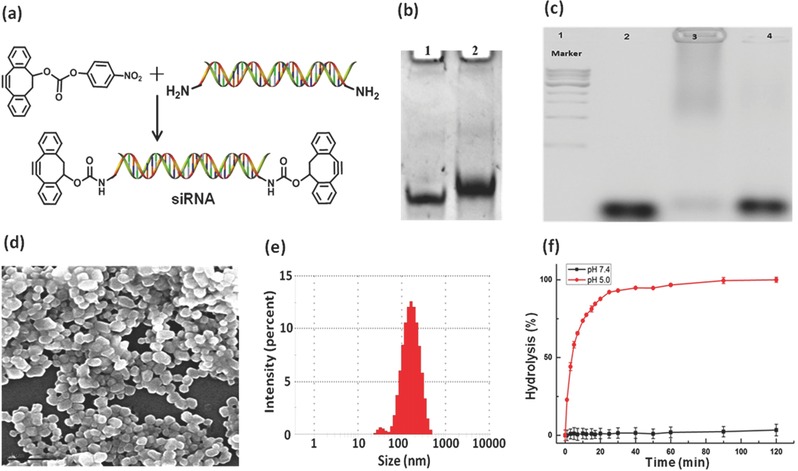
a) Synthetic route of cyclooctyne modified siRNA. b) PAGE gel verification of successful modification of siRNA. Lane 1: free siRNA; Lane 2: cyclooctyne modified siRNA. c) Agarose gel electrophoresis assay for the release of siRNA by acid induced hydrolysis. Lane 2: cyclooctyne modified siRNA duplex; Lane 3: PNSDS; Lane 4: acid hydrolysis of PNSDS. d) SEM image of PNSDS. Scale bar is 1 μm. e) Particle size of PNSDS. f) The hydrolysis of AAPEG at pH 7.4 and 5.0.

To investigate the potency of PNSDS to facilitate the siRNA delivery, tumor necrosis factor alpha (TNF‐α) siRNA was chosen to fabricate PNSDS, and its ability to alleviate inflammation‐induced cell damages was evaluated. As shown in the **Figure** **3**a, sense strand of TNF‐α siRNA, bearing primary amine groups at the both terminals, reacts with 4‐nitrophenyl chloroformate activated cyclooctyne,^32^ generating cyclooctyne functionalized TNF‐α siRNA (COT‐siRNA). The efficacy of the conjugation reaction was examined by RNA gel electrophoresis experiments, and Figure 3b shows that siRNA showed a clear gel shift after modification, indicating the completion of the reaction. The COT‐siRNA was purified by dialysis, and the successful modification is evidenced by Mass spectrum analysis. For example, MS data show that in comparison of TNF‐α siRNA with molecular weight of 16618.15, COT‐siRNA has a molecular weight of 17109.70, indicating the successful preparation of cyclooctyne functionalized TNF‐α siRNA.

An ideal nanoparticle based siRNA delivery system is expected to have some common structural features including a small particle size (10–200 nm) and high amount of siRNA loading. Scanning electron microscopy (SEM) was performed to examine the morphology and size of PNSDS. Figure 3d demonstrates that PNSDS has a well‐dispersed spherical morphology with an average size of 146 nm (Figure 3e) and zeta potential of −18.2 mV, indicating that the rapid crosslinking via SPAAC is well suited for the one‐pot construction of well‐defined nanoparticles. In addition, gel experiments show that almost all of the free siRNAs were incorporated into the nanoparticles (shown in Figure 3c), indicating that PNSDS has high siRNA loading capacities and further enhance the siRNA therapeutic potency.

PNSDS is formed by crosslinking of siRNA with acid labile acetal linkages on the surface of AAPEG, which is designed to be stable in the physiological environment with pH 7.4, but allows to release siRNA from PNSDS in response to acidic intracellular environment (pH 4.5–6.5). Although extensive crosslinking of PNSDS prevents nuclease degradation of siRNA, it also suppresses the release of siRNA from complexes. Therefore, pH sensitive hydrolysis of acetal linkages in PNSDS is a critical factor for the successful delivery of siRNA. To verify the hypothesis, the hydrolysis kinetics of AAPEG was measured at pH 5.0 and 7.4. Figure 3f demonstrates that AAPEGs is stable at pH 7.4, but undergoes rapid hydrolysis at pH 5.0 with an estimated half‐life of 10 min, indicating that AAPEG has a pH stimuli‐responsive property. In addition, we performed extra experiments on PNSDS to determine its acid‐degradability and ability to release siRNA from PNSDS in acidic conditions, via gel electrophoresis. Figure 3c shows that PNSDS underwent rapid hydrolysis under acidic conditions of pH 5.0, and released conjugated siRNA (Lane 4), indicating that PNSDS is a promising candidate for facilitating the transportation of siRNAs in response to intracellular acid environments.

### 2.3 Cellular Uptake and Cytotoxicity of PNSDS

**Figure 4:**
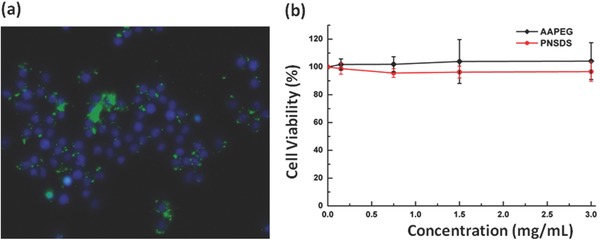
a) Fluorescence images showing internalization of PNSDS‐FAM‐siRNA‐in RAW 264.7 cells. b) Cell viability results after incubation of macrophage cells with various concentrations of AAPEG and PNSDS.

The above results have demonstrated that siRNA can be easily incorporated into AAPEG and form well‐defined nanocomplexes. We next examined whether PNSDS can carry siRNA into cells. Macrophage cells (RAW 264.7), in which nucleus was stained with 4′,6‐diamidino‐2‐phenylindole, were incubated with PNSDS carrying FAM labeled siRNA, and the uptake and intracellular location of siRNA were investigated by fluorescent microscopy. **Figure** **4**a shows that siRNA was widely distributed in the cytoplasm after endocytic internalization, indicating that PNSDS can successfully deliver siRNA into cells without the aid of any kind of positive charge which is one of the main obstacles hampering the clinical translation of siRNA.

Since the toxicity of the delivery material is the major hurdle that hinders the use of siRNA as therapeutics, we tested the cytotoxicity of a series of different concentrations of AAPEGs and PNSDSs with MTT assay. Since we did not introduce any positive charges to AAPEGs, we expected that the materials should have good biocompatibility. As anticipated, both AAPEG and PNSDS showed minimum cytotoxicity even at the highest concentration of 3 mg mL^−1^ (Figure 4b), suggesting that PNSDS provides a safe and effective approach for introducing siRNA into cells of interest.

### 2.4 In Vitro RNA Silencing with PNSDS

**Figure 5:**
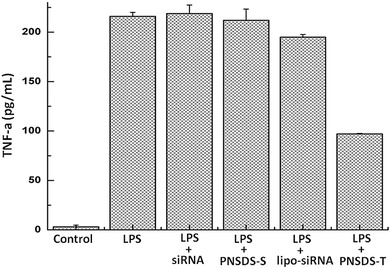
Knock down of TNF‐α produced by LPS in the presence of serum.

Next, we investigated whether the PNSDS could deliver functional siRNA into macrophages and inhibit gene expression. siRNA targeting TNF‐α was incorporated into the PNSDS and their ability to inhibit TNF‐α production in macrophages stimulated with lipopolysaccharide (LPS) was investigated.^33^ The macrophages were pretreated for 4 h with different formulations of siRNA at a concentration equivalent to 15 μg mL^−1^ of siRNA, including free TNF‐α siRNA, lipofectamine loading TNF‐α siRNA (lipo‐siRNA), PNSDS loading TNF‐α siRNA (PNSDS‐T), PNSDS loading scramble siRNA (PNSDS‐S) as a negative control. And then, all cell groups were treated with 100 ng mL^−1^ of LPS for 20 h to stimulate TNF‐α production, which was quantified using an enzyme linked immunosorbent assay (ELISA). **Figure** **5** demonstrates that PNSDS‐T can efficiently inhibit the expression of TNF‐α in macrophages. The expression of TNF‐α in macrophages pretreated with PNSDS‐T under oxidative stress decreased down to 50%, whereas free TNF‐α siRNA transcripts hardly suppressed TNF‐α production in macrophages because of its rapid nuclease degradation (Figure S2, Supporting Information) and low cellular uptake. Noticeably, lipofectamine only had slight silencing effect, and PNSDS‐S pretreated cells, as a negative control, did not show any decrease of amounts of TNF‐α expression at the indicated incubation times. The experiments confirm that PNSDS are able to efficiently deliver TNF‐α siRNA, selectively initiate gene silencing, and protect cells against inflammation‐induced damages.

### 2.5 Targeted PNSDS for In Vivo Gene Silencing and Reversing Inflammation Induced Liver Injury

To achieve the therapeutic applications of siRNA, the delivery systems not only need to protect siRNA against serum nucleases, prevent renal clearance, stimulate endosomal disruption, enhance cellular uptake, and release siRNA into cytoplasm, but also need to selectively localize and target siRNA to the diseased tissue. Therefore, an ideal nanoparticle based siRNA delivery system should have the multifunctional properties, which can be accomplished by optimizing the cross‐linking density, surface‐active, stimuli‐responsive constituents, and targeting moieties associated with specific diseases. We have demonstrated that well‐dispersed spherical PNSDS could be easily prepared via SPAAC methods. We further utilized SPAAC strategy to functionalize the PNSDS with the mannose moieties, which can improve its ability to target macrophages and to minimize aggregation with serum proteins. Mannose moieties were introduced in PNSDS via mixing cyclooctyne functionalized mannose with PNSDS‐T or PNSDS‐S to block the residual azides on AAPEG, leading to mannose modified PNSDS (M‐PNSDS‐T or M‐PNSDS‐S).

**Figure 6:**
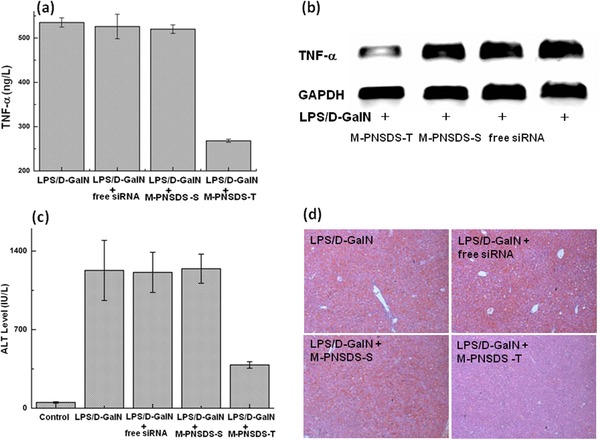
Mannose conjugated siRNA cross‐linked nanoparticles protected mice from LPS/d‐GalN‐induced acute hepatic injury. a) Serum TNF‐α level of mice administered with siRNA at 50 μg kg^−1^. b) Relative TNF‐α mRNA levels in mouse liver. c) ALT levels in mice 6 h after LPS/d‐GalN stimulation. d) H&E stained liver sections from mice 6 h post LPS/d‐GalN stimulation. *n* = 4.

We further investigated the potential of M‐PNSDS‐T to mediate TNF‐α gene silencing and treat the inflammatory liver diseases, which is one of major causes of mortality still increasing year‐on‐year. Acute liver failure, induced in mice by LPS and d‐galactosamine (d‐GalN), was chosen as the animal model, because LPS can stimulate macrophages to release inflammatory mediator TNF‐α, which plays a major role in the development of liver failure.^34^, ^35^ The mice were pretreated with TNF‐α siRNA, M‐PNSDS‐T, or M‐PNSDS‐S at a dose equivalent to 50 μg kg^−1^ of siRNA via the intraperitoneal injection. 24 h later, these mice were subsequently challenged with an intraperitoneal injection of LPS/d‐GalN. The therapeutic efficacy of PNSDS for acute liver failure was then evaluated by measuring the expression levels of TNF‐α in the serum. **Figure** **6**a shows that compared to mice only challenged with LPS/d‐GalN, mice pretreated with M‐PNSDS‐T had a twofold decrease in TNF‐α level after challenged with LPS/d‐GalN. In contrast, neither free TNF‐α siRNA nor M‐PNSDS‐S had inhibitory effect on the production of TNF‐α. Additionally, liver tissue was isolated from these mice and the expression of TNF‐α mRNA in liver was determined by gel electrophoresis to determine the specificity of PNSDS mediated gene suppression. Figure 6b shows that M‐PNSDS‐T significantly knock down the expression of TNF‐α mRNA in the liver in comparison of free TNF‐α siRNA and M‐PNSDS‐S, indicating that PNSDS can selectively deliver TNF‐α siRNA into liver macrophages, inhibit TNF‐α production efficiently, and minimize off‐target effects of siRNA.

Finally, we investigated the potential of PNSDS to protect mice from inflammation induced acute hepatic injury. Liver injury was estimated by biochemical serum markers such as alanine transaminase (ALT) and liver histopathological examination. Figure 6c shows that prior exposure of mice to M‐PNSDS‐T greatly modulates host response to LPS/d‐GalN challenge. For example, mice pretreated with M‐PNSDS‐T exhibited significantly lower serum ALT than mice with the pretreatment of free TNF‐α siRNA or M‐PNSDS‐S. In addition, histological examination on HE‐stained liver sections further confirmed the remarkable therapeutic efficacy of PNSDS. In comparison of healthy liver with normal histology, liver tissue in LPS/d‐GalN + M‐PNSDS‐T group, LPS/d‐GalN + M‐PNSDS‐S group, and LPS/d‐GalN + free TNF‐α siRNA group showed different levels of injury (Figure 6d). Mice receiving M‐PNSDS‐T showed significantly less liver histopathological damage than the other two groups with pretreatment of M‐PNSDS‐S or free TNF‐α siRNA, which had serious liver damage including congested central vein, disarranged hepatocytes, and broken cytolemma. Therefore, M‐PNSDS‐T shows robust therapeutic efficacy in the treatment of d‐GalN/LPS induced liver damages, indicating that PNSDS has a great potential as effective siRNA therapeutics for the treatment of acute liver failure.

## 3 Conclusions

In summary, we have developed a novel pH‐responsive nanoparticle based siRNA delivery system, termed the PNSDS, for selective delivery of siRNA both in vitro and in vivo. PNSDS has a unique crosslinking structure, which allows it address all challenges of siRNA delivery including the perspectives of physicochemical properties of siRNA, pharmacokinetics and biodistribution, and intracellular trafficking. We show that PNSDS is capable of delivering TNF‐α siRNA into macrophage cells, and induces efficient and specific down regulation of the expression of TNF‐α. In addition, mannose functionalized PNSDS exhibits efficient TNF‐α siRNA gene silencing in vivo, and displays remarkable anti‐inflammatory effects against hepatic injury. Furthermore, PNSDS can be simply prepared by crosslinking of AAPEG with siRNA crosslinker carrying concyclooctyne moieties via SPAAC, indicating that PNSDS provides an efficient and modular platform for systemic delivery of various siRNA by utilizing different siRNA sequence. Therefore, the PNSDS provides a clinically suitable, safe, and effective siRNA delivery system, and can be broadly used in RNAi therapeutics for disease prevention and treatment.

## 4 Experimental Section

Materials, characterization, detailed synthetic procedure of AAPEG and targeting group, and primer sequences for RT‐PCR are described in the Supporting Information.

*Synthesis of Cyclooctyne Modified siRNA*: 4 × 10^−3^ m (10 μL) of siRNA modified with an amine group at both ends in DEPC water was reacted with 30 × 10^−3^ m (30 μL) of 4‐nitrophenyl chloroformate activated cyclooctyne, a functional group for copper free click, dissolved in dimethyl sulphoxide (DMSO). After 4 h incubation at room temperature, the residual and byproduct was eliminated through dialysis in deionized water for overnight. The resulting product was stored at −20 °C for further use. The complete and successful reaction of siRNA with cyclooctyne was confirmed by gel shit assay and mass spectrum.

*General Procedure for the Preparation and Characterization of siRNA Crosslinked Particles*: 1 μL of this duplex solution (10 × 10^−6^ m) was mixed with 2.5 μL of AAPEG (200 × 10^−6^ m), and kept at room temperature for 10 min. The successful incorporation of siRNA to the nanocomplexes (PNSDS) was confirmed by agarose gel and characterized for size and zeta potential using dynamic light scattering (DLS, Zetasizer Nano‐ZS, Malvern), and also characterized by SEM (4800, Hitachi, Japan). Mannose conjugated nanoparticles (M‐PNSDS) was formed by simply mixing PNSDS with cyclooctyne modified mannose (compound **4** shown in the Supporting Information) and the size was characterized by DLS (Figure S3, Supporting Information).

*Hydrolysis of AAPEG*: A stock solution of AAPEG (2.2 mg per 40 μL) in phosphate‐buffered saline (PBS) solution (pH = 8.0) was prepared and 2.5 μL (3.35 × 10^−3^ m) AAPEG was added to a 700 μL PBS solution at either pH 5.0 or 7.4, in a spectrophotometer cuvette. The hydrolysis of the acetal was monitored at 37 °C by measuring the absorbance of at 280 nm.

*The Release of siRNA from PNSDS*: Gel electrophoresis was performed to determine if free siRNA could be released from the acid degradable nanocomplexes. A small amount of PNSDS was hydrolyzed for 2 h at pH 5.0 and the resulting solution was run on a 1% agarose gel at 100 V for 15 min. After that, a photograph was taken with a gel imaging system.

*Fluorescent Microscopy of siRNA Delivered with PNSDS*: This study investigated the intracellular distribution of siRNA delivered by PNSDS to macrophages, using fluorescent microscopy. RAW264.7 macrophages were maintained at 37 °C under a humidified atmosphere of 5% CO_2_ in Dulbecco's Modified Eagle's Medium (DMEM) containing 10% (v/v) fetal bovine serum (FBS), supplemented with penicillin (100 U mL^−1^) and streptomycin (100 mg mL^−1^). The macrophages (2 × 10^5^ cells per well, 24‐well plate) were incubated with PNSDS with FAM‐siRNA as cross linker for 4 h. Cells were washed three times with ice cold PBS, then fixed with 4% formaldehyde for 10 min, washing again with PBS for two times, adding 5 μg mL^−1^ of DAPI solution to the fixed cells for 5 min, the cells were washed three times with PBS and imaged with fluorescent microscopy.

*Delivery of TNF‐α siRNA with the PNSDS In Vitro*: RAW264.7 macrophages (1 × 10^5^ cells per well, 96‐well plate) in DMEM with 10% FBS were incubated with either PNSDS TNF‐α siRNA, PNSDS‐scrambled siRNA or free TNF‐α siRNA for 4 h (all samples had 15 μg mL^−1^ of siRNA). The cells were stimulated with 100 ng mL^−1^ LPS for another 20 h to induce TNF‐α. The amount of extracellular TNF‐α production was determined using an ELISA assay kit following the manufacturer's instructions in the kit.

*Cytotoxicity of the AAPEG and PNSDS (MTT Reduction Assay)*: An MTT reduction assay was performed to measure the cytotoxicity of the AAPEG and PNSDS. The macrophages (1 × 10^5^ cells per well, 96‐well plate) were treated with the AAPEG and PNSDS at various concentrations (0.075–3 mg mL^−1^) for 24 h. After pouring out the medium, the cells were then treated with 100 mL of MTT (1 mg mL^−1^ in PBS) and incubated for another 4 h. The medium was removed, the cells were lysed by adding 150 μL of DMSO, and the absorbance of the purple formazan was recorded at 520 nm using a microplate reader (Berthold TriStar LB 941, Germany). Percentage cell viability was calculated by comparing the absorbance of the control cells to that of AAPEG and PNSDS treated cells respectively.

*M‐PNSDS Induced In Vivo RNAi against Liver Injury*: Male C57BL/6 mice were intraperitoneally injected with free siRNA, M‐PNSDS containing TNF‐a siRNA or Scr siRNA at a siRNA dose of 50 μg kg^−1^ (4 mice per group), and with untreated mice serving as a control group. Twenty‐four hours post administration, LPS/d‐GalN (12.5 mg kg^−1^ and 1.25 g kg^−1^) were intraperitoneally injected. Blood was collected 2 h later to determine the serum TNF‐α level by ELISA (eBioscience, USA).

In another experiment, mice were administered with free siRNA, M‐PNSDS containing TNF‐a siRNA or Scr siRNA at a siRNA dose of 50 μg kg^−1^ (4 mice per group), and i.p. challenged with LPS/d‐GalN as described above. Six hours later, blood was collected and serum was isolated via centrifugation. Serum ALT levels were determined by clinical laboratory of XinQiao Hospital (Third Military Medical University, China)). Mice were then sacrificed; liver was harvested, cut into small pieces, washed with saline, and homogenized with RNAiso Plus (Takara Biotechnology Co. Ltd, China) reagent to extract the total RNA. cDNA was synthesized from 1 μg total RNA with PrimeScriptTM II1st Strand cDNA Synthesis kit (Takara Biotechnology Co. Ltd, China) according to the manufacturer's suggested protocol. The reaction condition was 42 °C for 60 min, 95 °C for 5 min. Then the synthesized cDNA was amplified. Primers used for the amplification of TNF‐α and the GAPDH gene were as in Table S1 (shown in supporting information). PCR was performed under the following conditions: 95 °C for 30 s, 55 °C for 30 s, and then 72 °C for 1 min (30 cycles). After that, 0.8% agarose gel was used to analyze the levels of the TNF‐α mRNA, the GAPDH as reference gene to normalize the data. For histological evaluation, mouse liver was harvested 6 h post LPS/d‐GalN stimulation, fixated in paraffin, cross‐sectioned, and stained with hematoxylin/eosin. Images were acquired by microscopy (Olympus IX 51, Japan).

## Supporting information

As a service to our authors and readers, this journal provides supporting information supplied by the authors. Such materials are peer reviewed and may be re‐organized for online delivery, but are not copy‐edited or typeset. Technical support issues arising from supporting information (other than missing files) should be addressed to the authors.

Supplementary
