# Supplementary material for: SiRNA Crosslinked Nanoparticles for the Treatment of Inflammation‐induced Liver Injury
Source: Adv Sci (Weinh). 2016 Sep 2;4(2):1600228. doi: 10.1002/advs.201600228 (PMC5323819; doi:10.1002/advs.201600228)
Supplement: Supplementary file 1 — Supplementary [file ADVS-4-na-s001.pdf]

Copyright WILEY-VCH Verlag GmbH & Co. KGaA, 69469 Weinheim, Germany, 2016.

ADVANCED SCIENCE
Open Access

Supporting Information

for *Adv. Sci.*, DOI: 10.1002/advs.201600228

SiRNA Crosslinked Nanoparticles for the Treatment of Inflammation-induced Liver Injury

Yaqin Tang, Ziying Zeng, Xiao He, Tingting Wang, Xinghai Ning, and Xuli Feng**

Supporting Information

SiRNA Crosslinked Nanoparticles for the Treatment of Inflammation induced Liver Injury

Yaqin Tang, Ziying Zeng, Xiao He, Tingting Wang, Xinghai Ning, and Xuli Feng**

Materials and Methods

Lipopolysaccharide (LPS, from E. coli serotype O55:B5) was bought from Sigma (St. Louis, MO, USA) and D-galactosamine (D-GalN) was purchased from J&K Scientific Ltd. (beijing, China). 8-armed PEG was purchased from Jenkem Technology Co., LTD (Beijing China). Amberlite IR120 H form ion-exchange resin and 1,2,3,4,6-penta-Oacetyl-a-D-mannopyranoside were purchased from J&K Scientific Ltd. (Beijing, China). Palladium-on-Carbon was bought from Adamas Reagent Co. Ltd. (Shanghai, China). Enzyme-linked immunosorbent assay (ELISA) kit (TNF-α ELISA Ready-Set-Go) for the detection of TNF-α was purchased from eBioscience (San Diego, CA, USA). Negative control siRNA with a scrambled sequence (scRNA, 5'- NH_2 - C6/ ACUACUUCUGCUGAGUCACUUUAAGUA /3'- NH_2 - C7 and antisense 5'-UAAAGUGACUCAGCAGAAGdTdT-3') and siRNA targeting TNF-α mRNA (5'-NH_2-C6/ACUAAGACAACCAACUAGUGGUGCUUAGUA/3'- NH_2 - C7 and antisense 5'-GCACCACUAGUUUGUCdTdT-3') were synthesized by GenScript Corporation (Nanjing, China). Other Nucleotide primers were purchased from Sangon Biotech Shanghai Co. Ltd. The ^{1}H-NMR and ^{13}C-NMR spectra were recorded on a Bruker Avance 400 MHz spectrometer. P-toluenesulfonic acid was recrystallized from toluene, molecular sieves were freshly dried right before use, other regents and solvents were used without further purification. All the water used in the experiments was DNase and RNase free.

Male C57BL/6 mice (6-8 week old) were purchased from XinQiao Hospital, Third Military Medical University (China). All mice were housed in a clean room with four mice per cage. Mice were given access to water, food and exposed to a 12:12 h light–dark cycle (8:00 am-8:00 pm) at 25 ± 1 °C. All study protocols were reviewed and approved by Laboratory Animal Welfare and Ethics Committee of the Third Medical University.

Synthesis of compound 2.

3-azidopropan-1-ol (12.16 g, 120.4 mmol, 4 equiv.), 5 A sieves (20 g), 4-(2-hydroxyethoxy)benzaldehyde (5 g, 30.1 mmol, 1 equiv.), and p-toluenesulfonic acid (0.84 g, 4.8 mmol) were dissolved in dry tetrahydrofuran (60 mL). The reaction mixture was left to stir overnight at room temperature. The reaction was then quenched with triethylamine (2 mL), after removing the sieves, the filtrate was concentraed by reduced pressure, then directly loaded onto a silica gel column. The column was eluted with a 1:5 ethyl acetate/hexane mixture and then a 1:1 ethyl acetate/hexane mixture to afford 4.5 g (42.8%) of the acetal. ^{1}H NMR (400 MHz, $CDCl^3$): δ 1.76 (m, 4), 3.34 (m, 4), 3.48 (m, 2), 3.56 (m, 2), 4.05 (m, 2), 4.1 (m, 2), 5.45 (s, 1), 6.9 (d, 2), 7.33 (d, 2), ^{13}C NMR ($CDCl_3$): δ 30.5, 48.5, 60.2, 61.5, 69.2, 101.59, 114.26, 127.89 (q, J = 143.7 Hz), 130.95, 158.86. LC-MS (m/z): $C_{15}H_{22}N_6O_4Na^+$ [M+Na], calcd, 373.18; found, 373.39.

Synthesis of compound 3.

2 grams of 2-(4-(bis(3-azidopropoxy)methyl)phenoxy)ethanol (5.7 mmol) was dissolved in anhydrous DCM, the reaction was then cooled to 0°C and triethylamine (2.6 mL) was added. Then acryloyl chloride (0.8 mL) diluting into 10 mL of DCM was drop added. The reaction mixture was left to stir overnight at room temperature. A 10:1 water/K_2CO_3 solution (200 mL) was added and the reaction was allowed to stir for 5 minutes before extracting the product into methylene chloride (3 x 200 mL). The organic layer was dried and evaporated to afford yellow oil. The oil was loaded onto a silica gel column and eluted with a 1:4 ethyl acetate/hexane mixture to afford 1 g (43.2%) of the product as colorless oil. ^{1}H NMR (300 MHz, $CDCl_3$): δ 1.89 (m, 4), 3.37 (m, 4), 3.49 (m, 2), 3.60 (m, 2), 4.21 (t, 2), 4.50 (t, 2), 5.46 (s, 1), 5.65 (d, 1), 6.12 (q, 1), 6.88 (d, 1), 7.25 (d, 2), 7.35 (d, 2). ^{13}C NMR ($CDCl_3$): δ 29.6, 48.2, 61.97, 62.87, 65.96, 101.59, 114.35, 127.90, 128.08, 128.49, 131.09, 158.69, 166.10. LC-MS (m/z): $C_{18}H_{24}N_6O_5H^+$ [M+H], calcd, 405.18; found, 405.40.

Synthesis of AAPEG.

To a stirred solution of **2** (700 mg, 1.73 mmol) in MeOH was added 8 armed PEG-NH_2 (300mg, MW: 15000). The mixture was stirred at room temperature for 24 hours under nitrogen. The reaction mixture was poured into large amounts of ice cold ether, the resulting solid was filtered and redisolved in $CHCl_3$, and repeated the precipitation process for three times to give the product as yellow white power. ^1H NMR (300 MHz, $CDCl_3$): δ 7.28 (s, 2), 6.86 (s, 2), 5.54 (s, 1), 4.38 (m, 3), 4.2-4.12 (m, 2), 3.8-3.37 (m, 90), 2.75-2.67 (m, 3), 2.47-2.40 (m, 2), 1.9-1.76 (m, 6).

Synthesis of cyclooctyne functionalized mannose

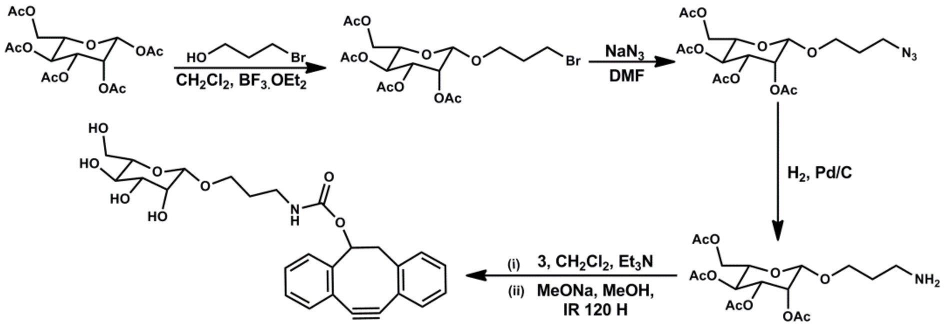

Figure S1. Synthetic routes of compound **4**

3-Aminopropyl-2,3,4,6-tetra-O-acetyl-a-D-mannopyranoside (0.162 g, 0.4 mmol) was added to a solution of Carbonic acid, 11,12- didehydro-5,6-dihydrodibenzo[a,e]cycloocten-5-yl 4-nitrophenyl ester (40 mg, 0.1 mm) and Et_3N (50 uL) in dry DCM (3 mL). The reaction was stirred at RT for 6 h. TLC analysis, using PE/ EA (1:1, v/v), showed that the reaction had gone to completion. The resulting oil was then purified using column chromatography on silica gel (ethyl acetate/hexane (1:1, v/v)). The relevant parts were collected, combined and concentrated to dryness under reduced pressure. The obtained product (47 mg, 0.072 mm) was then dissolved in dry MeOH (2 mL), including sodium methylate (1 M, 20 uL). After completion of the reaction by TLC, the solution was neutralized with the Amberlite IR120 H form, ion-exchange resin. The resin was then filtered and the filtrate was concentrated to yield the target molecule (31mg, 91%). ^1H NMR (400 MHz, $(CD_3)_2SO$) 7.34-7.63 (m, 8), 7.23-7.26 (dd, 1), 7.07 (s, 1), 5.27 (m, 1), 4.53-4.60 (m, 2), 3.56-3.64 (m, 3), 3.25-3.43(m, 7), 4.82 (d, 1), 2.96-3.09 (m, 2), 2.70-2.76 (m, 2), 1.66-1.71 (m, 2). ^{13}C NMR (400 MHz,

$(CD_3)_2SO$): δ=155.64, 152.84, 151.32, 130.63, 128.68, 127.95, 126.57, 126.24, 124.37, 123.33, 120.78, 112.88, 110.36, 100.23, 75.72, 74.45, 71.45, 70.66, 67.25, 64.25, 61.67, 64.26, 61.67, 55.46, 45.91, 38.14, 29.484. ESI-MS (m/z): $C_{26}H_{29}NO_8Na^+$ [M+Na], calcd, 506.1893; found, 506.1790.

Table 1. TNF-α and GAPDH primer sequences.

Primer	Sequence
TNF-α F	CCACCACGCTCTTTCTGTCTACTG
TNF-α R	GGGCTACAGGCTTGTCACTCG
GAPDH-F	TGATGACATCAAGAAGGTGGTGAA
GAPDH-R	TCCTTGGAGGCCATGTAGGCCAT

Enzyme stability assay

PNSDS and free siRNA were incubated separately with 10% FBS at 37°C for 1h. The FBS was inactivated by heating at 80°C for 5 min. Finally, protected siRNA was released from PNSDS by lowering the pH to 5 at 37°C for 30 min. Each sample was detected by resolving on a 1% agarose gel (with 0.1 mg/ml EtBr) in 0.5 x TAE running buffer at 90 V for 20 min respectively.

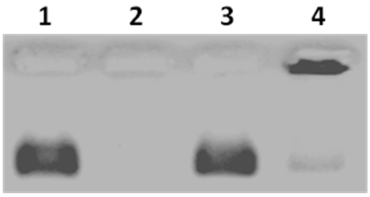

Figure S2. Characterization of stability of PNSDS by gel electrophoresis. Lane 1, free siRNA; lane 2, siRNA + FBS; lane 3, PNSDS + FBS after acid release; lane 4, PNSDS.

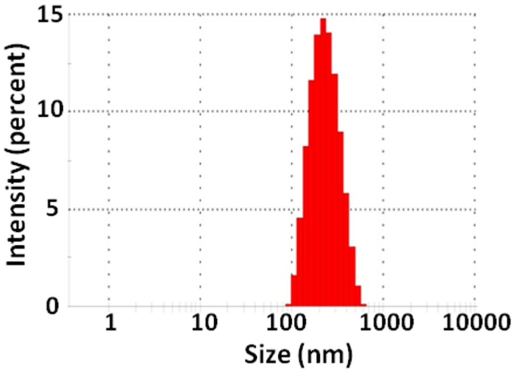

Figure S3. Particle size of M-PNSDS.
